# Evaluation of anxiety and depression in patients with knee osteoarthritis

**DOI:** 10.1192/j.eurpsy.2024.731

**Published:** 2024-08-27

**Authors:** A. Feki, I. Sellami, I. Mnif, Z. Gassara, S. Ben Djemaa, A. Abbes, M. Ezzeddine, M. H. Kallel, H. Fourati, R. Akrout, S. Baklouti

**Affiliations:** ^1^Rheumatoloy, Hédi Chaker Hospital; ^2^ Medicine univeristy; ^3^Occupational medicine, Hédi Chaker Hospital, Sfax, Tunisia

## Abstract

**Introduction:**

Knee osteoarthritis is one of the most common causes of functional impairment, significantly impacting patients’ quality of life and leading to severe mood disorders. Our objective is to assess the prevalence of depression and anxiety in knee osteoarthritis patients.

**Objectives:**

Evaluate the prevalence of depression and anxiety in patients with knee osteoarthritis.

**Methods:**

This was a cross-sectional study conducted over a three-month period from February to April 2022, including consecutive patients who consulted in a Rheumatology department. We assessed each patient using a validated version of the HAD (Hospital Anxiety and Depression) scale, which includes 14 items, each rated from 0 to 3, measuring two components: depression and anxiety.

**Results:**

We enrolled 82 patients (67 women and 15 men) with an average age of 60.4 years [44-89 years]. The average disease duration was 10 years [2-30]. Knee osteoarthritis was bilateral in 79% of cases. Knee deformities were observed in 74.4% of cases (40.2% had genu valgum, and 29.3% had genu varum). Radiological assessment showed that most of our patients were at Kellgren-Lawrence (KL) stage 3 (50%). All patients received analgesics, with 92.7% receiving NSAIDs, 67.1% local corticosteroid infiltrations, and 18.3% hyaluronic acid injections. The mean visual analog scale (VAS) score was 6.9 out of 10 [1-10]. The mean anxiety score was 7.5 [4-16], with 25.4% of patients exhibiting no anxiety symptoms (score ≤7), 40.3% displaying doubtful anxiety symptomatology (score between 8 and 10), and 34.3% having certain anxiety symptomatology (score ≥11). The mean depression score was 9.6 ± 4 [0-19]. 40% of patients had no depressive symptoms (score ≤ 7), 53.3% had doubtful depressive symptoms (score between 8 and 10), and 6.7% had certain depressive symptoms (score ≥11). The statistical analysis revealed a significant association between anxiety scores and KL stage, but no association with age, sex, mobility limitation, or VAS. Regarding depression, there was no significant association with epidemiological, clinical, or radiological parameters of knee osteoarthritis.

**Conclusions:**

Although knee osteoarthritis may appear to be a benign pathology, its impact can be severe, including depression and anxiety. These mood disorders are primarily influenced by the disease stage. Therefore, psychological care is sometimes necessary in the management of these chronic degenerative diseases.

**Disclosure of Interest:**

None Declared

